# Direct Printed Silver Nanowire Strain Sensor for Early Extravasation Detection

**DOI:** 10.3390/nano11102583

**Published:** 2021-09-30

**Authors:** Hsuan-Chin Lu, Ying-Chih Liao

**Affiliations:** Department of Chemical Engineering, National Taiwan University, No. 1, Sec. 4, Roosevelt Road, Taipei 10617, Taiwan; b06504022@ntu.edu.tw

**Keywords:** wearable sensor, nanowire alignment, extravasation detection, percolation theory

## Abstract

In this study, we presented a wearable sensor patch for the early detection of extravasation by using a simple, direct printing process. Silver nanowire (AgNW) ink was first formulated to provide necessary rheological properties to print patterns on flexible plastic sheets. By adjusting printing parameters, alignments of AgNWs in the printed patterns were controlled to enhance the resistance change under stretching conditions. A resistive strain-sensing device was then fabricated by printing patterned electrodes on a stretchable film for skin attachment. The designed sensor pattern was able to detect forces from a specific direction from the resistance change. Moreover, the sensor showed excellent sensitivity (gauge factor (GF) = 100 at 50% strain) and could be printed in small dimensions. Sensors of millimeter size were printed in an array and were used for multiple detection points in a large area to detect extravasation at small volumes (<0.5 mL) at accurate bump location.

## 1. Introduction

Extravasation is the leakage of intravenously (IV) infused medication into the extravascular tissue surrounding the site of infusion due to the incorrect placement of cannula, excessive movement, or fragile veins. Depending on the drug’s properties and the leakage amount, extravasation can result in consequences ranging from local irritation to tissue necrosis. Studies in pediatric hospitals find extravasation injuries with a quoted rate of around 10% in children receiving IV medications [1,2]. A significant proportion of extravasations remains unreported since most incidents do not end up with significant clinical consequences. Those minor symptoms of extravasation, including redness or swelling, can still cause anxiety and distress, leading to medical disputes or patient dissatisfaction. Currently, prevention of extravasation injuries relies heavily on medical professionals to carefully administer infusions and constantly inspect the cannula site visually. For patients that receive continuous or intermittent infusions over a prolonged period of time, it is almost impossible for medical personnel to constantly observe or inspect the occurrence of extravasation. As a result, extravasation is frequently detected when a large subcutaneous “bump” or red swelling is noticed at the IV cannula site. At this stage, a large amount of infused fluid has extravasated and might lead to tissue necrosis or other serious complications. Thus, efficient methods of early extravasation detection are needed not only to protect against injuries, but also to prevent long-term functional and cosmetic sequelae.

With recent scientific advancements, wearable electronic devices have been designed for the early detection of extravasation. Goodman et al. [3] designed an electrode patch to measure the change in tissue impedance based on the ionic and non-ionic contrast. After attachment to skin, drug leakage around the cannula site results in ionic impedance change and thus extravasation can be quickly detected. Similarly, by directly incorporating a strain sensor in a wearable patch, Cheng et al. [4] showed the possibility of detecting small bumps 2 mm in height on human skin. To further improve the detection accuracy, Lee et al. [5] integrated pressure, temperature, and optical sensors into a wearable sensor system, and showed that the accuracy of extravasation detection can be largely increased to 83.7%. However, these sensing devices usually involve complicated fabrication processes. Moreover, these sensors are made of metals and thus obscure the site of cannulation, impeding direct observation by medical professionals or patients. Therefore, more research is still needed to investigate the possibility of fabricating an invisible strain sensor with a low cost for extravasation.

Printed sensors have received much attention in recent years due to their potential in many applications [6], including health monitoring [7,8], robotics [9,10], and electronic devices [11,12]. By using printing technologies, such as inkjet printing, screen printing, and direct ink writing, one can easily fabricate sensing devices for chemical or motion detections. A good example of such devices are strain sensors based on strain-induced changes in resistivity. These resistive strain sensors are usually fabricated by printing conductive nanowires or nanotube solutions, which usually contain polymeric binders, on a stretchable substrate. Recent progress in Ag nanoparticle-based sensors fabricated by selective laser sintering also present an interesting way to fabricate wearable strain sensors that have good sensing properties [13,14]. Because the connections of percolating nanomaterials are very sensitive to deformations, a large change in electric resistance, which is correlated directly with the contact resistances between conductive nanomaterials [15,16], can be observed upon small applied strain on the printed patterns. Among many conductive nanomaterials, silver nanowires (AgNWs) [17,18,19] have been shown to produce percolation networks capable of strain detection. Moreover, with the large aspect ratio, AgNWs are commonly used to produce transparent conductive patterns for sensors. By using various templating techniques, such as capillarity [20], ice-templating [21], and programmable brushing [22], the AgNW alignments can be well controlled and the sensitivity of the AgNW strain sensors can be much improved. However, those techniques are either complicated or time consuming. Another challenge for strain-sensing devices is the ability to differentiate strain directions from external touch. To address this problem, printed sensing devices with customized electrode array designs have been shown to be effective to determine the directions [23]. Therefore, a sensing array was also developed in this study to determine the location and off-plane force of the extravasation site.

In this study, we present a wearable sensor patch fabricated by a simple direct printing process for early detection of extravasation. AgNW inks with good printability were first formulated for conductive pattern fabrication. The resistivity, or the AgNW alignments in the printed patterns, were then adjusted by controlling the printing parameters. By taking advantages of the significant increase in contact resistance between AgNWs, the printed patterns can detect strain variation by measuring the resistance changes when the sensors are stretched. The sensitivity and detection limit of this resistive-type sensor were examined to evaluate their feasibility to be applied to early extravasation detection.

## 2. Materials and Methods

The silver nanowire suspension (1 wt% AgNW in water) was purchased from Cambrios Technologies Corp. Polyethylene oxide (PEO, MW = 2,000,000) was purchased from Sigma-Aldrich. The prepared AgNW inks were printed by using a dispensing system (DT-200F, Dispenser Tech Co., Ltd., New Taipei City, Taiwan). A nozzle with an inner diameter of 0.08 mm (34G) and a 3 mL syringe were mounted on the dispensing system to print the inks on flexible silicone sheets (Compo-SiL Skin, General Silicones, Hsinchu City, Taiwan). The printed electrodes were then dried in an oven at 60 °C for an hour. The electrical resistance of the prepared sensors was measured using a multimeter (HILA DM-2610, HILA, Taipei City, Taiwan). Viscosity measurements were obtained with a rheometer (Discovery HR-2, TA instruments, Taipei City, Taiwan). The surface morphology for the printed sensors was observed by scanning electron microscopy (SEM, Nova^TM^ NanoSEM 230, FEI, Hsinchu City, Taiwan).

## 3. Results and Discussion

### 3.1. Ink Formulation

**Figure 1:**
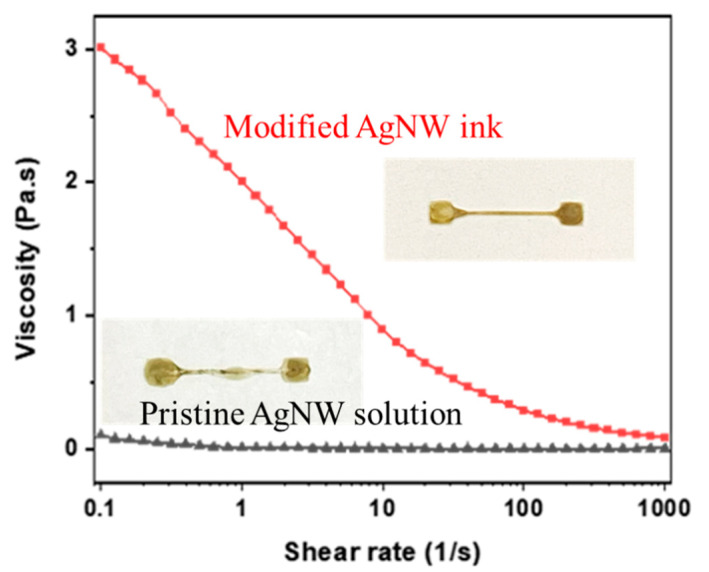
Shear thinning behavior of modified silver nanowire (AgNW) ink. The inset pictures are the printed patterns from the inks.

The AgNW inks were first formulated to print patterns with reproducible properties. The pristine AgNW suspension contained mostly water and thus possessed high surface tension (60 mN/m) and low viscosity (1 cP). Direct printing of the AgNW suspension led to undesirable outcomes, such as fluid-thread breakup and uneven boundaries. To avoid this problem, the ink was re-formulated. AgNWs were first centrifuged and washed from the pristine inks. The proper amount of clean AgNWs was then added to an aqueous solution containing PEO, ethanol, and water at a weight ratio of 0.03:1:1. The addition of ethanol lowered the ink surface tension (40 mN/m) and improved the wetting ability on the silicone substrate. The addition of PEO helped disperse the AgNWs because its hydroxy groups could bind with the surface of the AgNWs [24]. Moreover, PEO, a commonly used thickening agent, increased the ink viscosity with shear thinning characteristics (Figure 1) to help preserve the pattern definition. During the printing process, the shear rate in the nozzle was high and quickly declined as the ink spread on the substrate. Thus, the increased ink viscosity after printing on the substrate limited the ink flowability after printing and therefore yielded much better printing results, as shown in the inset pictures of Figure 1.

### 3.2. AgNW Concentration

**Figure 2:**
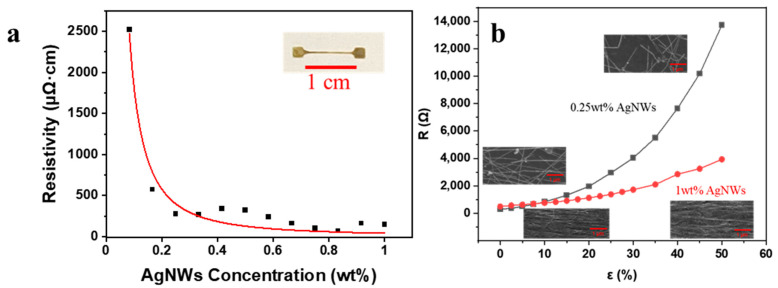
(**a**) The relationship between AgNW concentration and electrical resistivity. All the printed samples were straight lines with 1 cm length as shown in the inset image. (**b**) The relationship between strain and resistance for different AgNW concentrations. The inset pictures are the SEM pictures of the printed lines before and after stretching.

To increase the sensitivity of the printed sensors to strain, the AgNW concentration in the ink needed to be optimized. When a percolating network of AgNWs is subjected to strain, the connections between the AgNWs is broken, which results in increasing resistance. To determine the best AgNW ink for strain sensors, the resistances (*R*) of printed straight lines at different AgNW concentrations were recorded (Figure 2a). A power law relationship could be obtained as follows:

(1) $$\;\rho =102.14\;{C_{A}}^{-1.111}$$

where *C_A_* is the concentration of AgNWs in wt% and *ρ* is the resistivity of 1 cm wire printed with the AgNW ink in μΩ·cm. The critical exponent of 1.111 indicates a 2D percolation network [16] in the printed patterns. As indicated in Figure 2a, the resistance decreased quickly with increasing AgNW concentration and remained nearly constant when the concentration was higher than 0.25 wt%. Because a pronounced change in resistance at low strain is desired for strain sensors [25], it was expected that the sensors made by inks below 0.25 wt% would yield much better sensitivity due to their vulnerable percolating networks [26,27]. However, it was found (Figure 2b) that the resistance of the printed lines from low AgNW concentrations (<0.25 wt%) changed too dramatically at stretching conditions and easily exceeded the maximum measurable value. Therefore, 0.25 wt% was selected as the optimal concentration and was used in the next sessions.

The surface morphology was also investigated by SEM for printed patterns from both high- and low-AgNW-concentration inks. For high AgNW concentrations (1 wt%), the AgNWs were well aligned along the printing direction. Thus, adjacent nanowires were only slightly pulled apart from each other after being stretched and the resistance increased ~10 times afterwards. On the other hand, for low AgNW concentrations (0.25 wt%), the stretching resulted in disconnections between AgNWs and led to a dramatic increase in resistance ~100 times at 50% strain. From these observations, in order to leverage a large increase in resistance for strain-sensing applications, 0.25 wt% AgNW solution was selected and used in the following sections.

### 3.3. Printing Speed and AgNW Alignments

**Figure 3:**
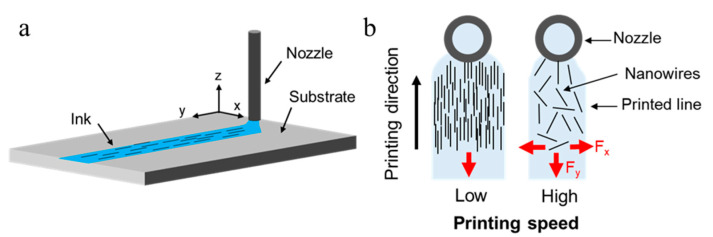
(**a**) Schematic diagram of nanowires during the nozzle extrusion process. (**b**) Effects of printing speed on AgNW alignment.

In addition to AgNW concentration, the percolation of AgNWs is also critical to the resistance of printed patterns. For parallelly aligned AgNWs, the contact between nanowires could be easily torn apart and cause more resistance change when submitted to strain. On the other hand, randomly distributed AgNWs showed better flexibility for percolating network connection and thus allowed the printed line to remain conductive even under large strain conditions [28]. Because the ratio between the length of AgNW (~30 μm) and the nozzle diameter (80 μm) was fairly close, the AgNWs oriented themselves in parallel when the solution passed though the long nozzle (~2 cm). As the ink exited the nozzle, the ink spread into liquid lines of different linewidths according to the ink flow rate (Figure 3). At a relatively high printing speed, the drag force caused by viscous flow resulting from the spreading of the ink on the substrate and the disturbance caused by the nozzle were significant. Therefore, the nanowires could rotate freely as the ink spread, and resulted in more random alignments. At lower printing speeds, lots of nanowires were deposited at a short distance. The large density prohibited the nanowires from rotating freely when drying on the substrate, and thus resulted in tightly packed, aligned nanowires in the printed line.

**Figure 4:**
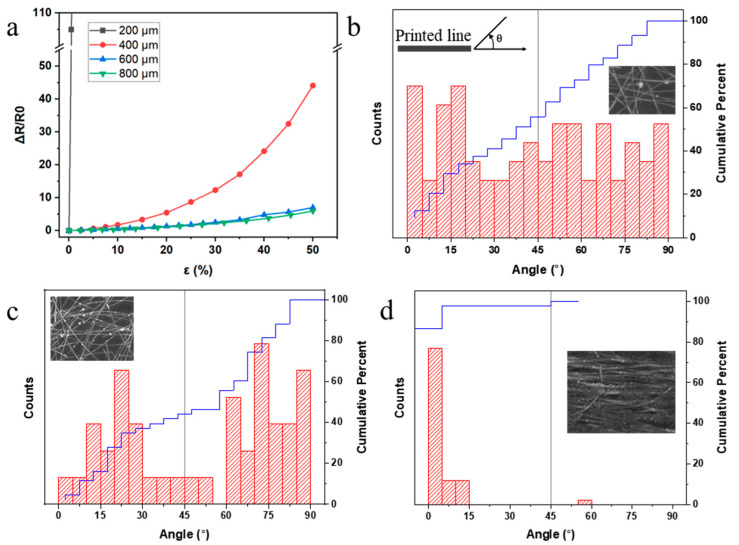
(**a**) Relationship between strain and normalized resistance change for sensors of different linewidths (w). The distribution of the AgNW alignment angle to the printing direction in printed lines with a width of (**b**) 200 μm, (**c**) 400 μm, and (**d**) 800 μm. Inset pictures are SEM images of the printed samples.

By using a constant ink flow rate, conductive lines with widths ranging from 200 to 800 μm were printed by reducing the printing speed. The electrical performances of these printed straight lines of 1 cm length with different linewidths were tested, and the relationships between strain and normalized resistance change ($\frac{\Delta R}{R_{0}}$) are shown in Figure 4a, where *R*_0_ is the resistance of an unstretched sensor. For the thinnest line (200 μm), i.e., the sample from the highest printing speed, a drastic change in resistance was observed when subjected to strain. However, the sample became non-conductive at ~1% strain without any measurable resistance, possibly due to the fragile AgNW network. After examining the SEM image of this sample (Figure 4b), it was found that the AgNWs were loosely distributed with random alignments. By performing image analysis using commercial software (ImageJ) on the SEM image, a flat distribution of the alignment angle was found. The random alignment with the low AgNW density led to fragile contact separation and caused the line to become nonconductive after a little strain was applied. When the linewidth was extended to 400 μm (Figure 4c) using a slightly higher printing speed, the AgNW network exhibited a bimodal distribution with peaks at 20° and 75°. A percolating network could be formed with suitable contact resistance for strain measurements. For the 800 μm linewidth, the nanowires were parallelly aligned and tightly packed together, resulting in good conductivity but little resistance variation in strain conditions, indicating low sensitivity in strain measurements. Thus, the printing parameters for the 400 μm linewidth were selected for strain sensor fabrication in the next sections due to the measurable resistance and high sensitivity.

### 3.4. Patterned Strain Sensor for Force Direction

**Figure 5:**
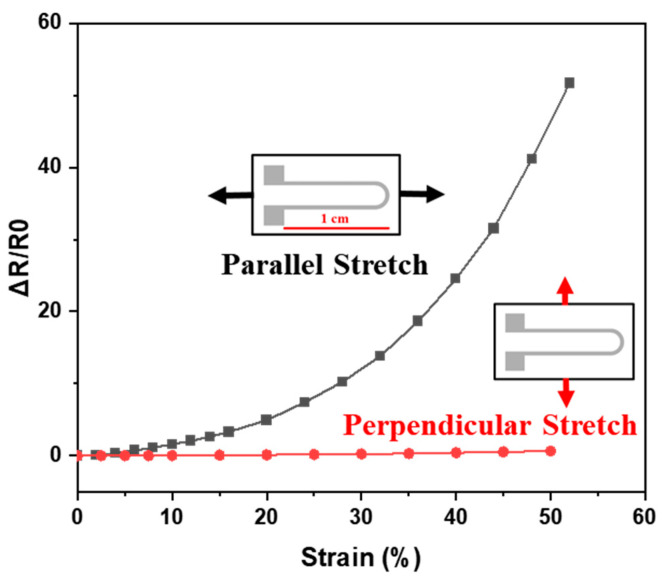
Resistive response of the printed sensor under parallel/perpendicular stretching directions.

To enable the printed sensor with 2D directional sensing abilities, the geometry design of the sensor is of critical importance. In this study, a hairpin shape with one side much longer than the other was used (Figure 5). As the sensor was stretched along the direction parallel to the long side, the strain led to a significant response. On the other hand, the sensor showed little to no response for strain in the perpendicular direction. This directional resistance variation originated from the fact that the resistance change was proportional to the length of the printed AgNW lines. The short length in the perpendicular direction means the perpendicular strain had less impact on the total resistance of the sensor, whereas the parallel direction strain could cause large resistance change quickly. To further characterize the printed strain sensor, the sensitivities were evaluated by calculating the gauge factor (GF):

(2) $$\mathrm{GF}=\frac{∆R/R_{0}}{∆L/L_{0}},$$

The gauge factor for the sensors ranged from approximately 10 at lower strains (~10%) to 100 at high strains (>50%). Compared to some reported flexible strain sensors (Table 1), our strain sensors exhibited much higher sensitivities due to the AgNW alignment optimization. This force directional differentiation allowed us to produce a sensing array with multiple sensors in alternating orientations. The printed sensor array could then be used to distinguish strains from different directions on a surface and provide us with an understanding of the 2D strain distribution on a given surface.

### 3.5. Extravasation Detection

**Figure 6:**
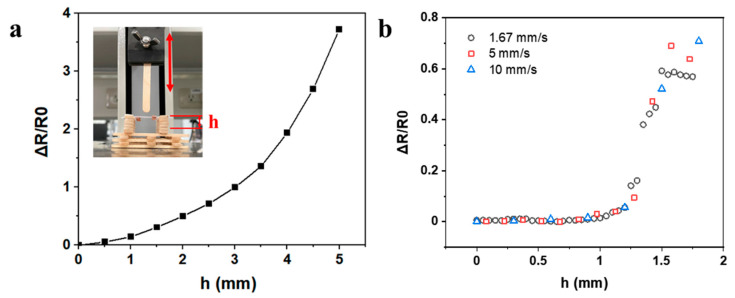
(**a**) The relationship between press distance h and relative resistance change of the sensor. (**b**) Resistive response of sensor under different pressing rates.

The hairpin shape strain sensor was also able to detect the out-of-plane forces. In previous sections, forces on the x–y plane caused strain and resistance changes. To evaluate the performance of the sensor for forces on the z-axis, the sensor was hung over a custom platform (Figure 6a) and pressed by a stick with a round tip. The resistance change of the sensor was recorded and plotted against the press distance *h*. As indicated in the figure, the resistance change was exponential to the press distance, and was similar to the results of a sensor under planar stretching. Besides the great sensitivity, the sensor also showed good response time to the bump formation or pressing without hysteresis. As shown in Figure 6b, the resistance responses under different pressing rates overlapped all together, indicating the strain detection was independent of the pressing speed. All these results indicate that the sensor exhibited good sensitivity and low detection limit regardless of the rate of bump forming, which is crucial for detecting all sorts of extravasation or bump formation at different liquid injection rates.

**Figure 7:**
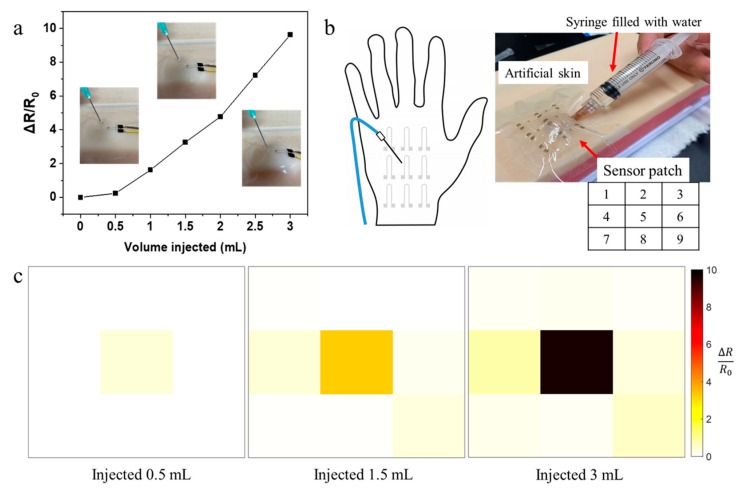
(**a**) The relationship between volume of water injected and resistance change. The inset pictures show the bumps after injection at the specific leak volume. (**b**) The apparatus for simulated extravasation: A sensor patch array (with the corresponding numbering of sensors) was attached to the artificial skin, under which water was injected to simulate extravasation. (**c**) Sensor response for the sensing array at three different extravasated volumes.

To evaluate the performance for extravasation detection, a sensor was attached to artificial skin with fake veins. Water was then injected near the vein to simulate extravasation. As the water volume increased, a bump gradually formed under the sensor, and the resistance change for the sensor was recorded and plotted against the injected volume of water (Figure 7a). As shown in the inset pictures, for a small bump on the artificial skin with little liquid volume (~0.5 mL), the sensor quickly detected the leakage with a relative resistance change of 25%. To further locate the extravasation site, a sensor array consisting of nine sensors was attached to the artificial skin (Figure 7b). As water was injected directly under the artificial skin near the middle of the sensor patch, the sensors responded quickly to the bump formation (Figure 7c). The sensor in the middle (sensor 5) clearly showed a much more significant response compared to the other sensors, locating the position of the extravasation. These results prove that the sensor could indeed detect extravasation at small volumes (<0.5 mL), and multiple sensors in an array could pinpoint the accurate location of extravasation sites. The sensors can be attached to body parts away from moving joints, such as the back of the hand or the forearm, so that human motion does not interfere with the extravasation signal.

## 4. Conclusions

A wearable sensor patch for the early detection of extravasation can be fabricated by using a simple, direct printing process to form conductive paths on a stretchable film, using an ink based on AgNWs. By adjusting the printing parameters, the number density and alignment of AgNWs can be modified to increase the resistance change of the printed AgNW line under stretching condition. By using a hairpin shape for the printed AgNW pattern, the printed sensor is able to detect forces from different axes with large gauge factor of 100 at 50% strain. Sensors of millimeter size can then be fabricated and allow the potential for multiple detection points in a large area. An array of sensors can be used for early extravasation detection of small leakage volumes (<0.5 mL) and accurately pinpoint the exact extravasation location. The sensor in this work combines easy fabrication with excellent sensing properties, which can also be applied to other applications, such as subtle human motion monitoring and other medical uses.

## Figures and Tables

**Table 1:** Comparison of flexible strain sensors.

Author	Materials	Gauge Factor (Strain)	Reference
Ali et al. (2018)	Silver nanowire/silver flake composite	22 (10%)	[17]
Kim et al. (2015)	Pre-strained silver nanowire percolation network	20 (35%)	[18]
Min et al. (2019)	Silver nanoparticle/MWCNT composites	58.7 (74%)	[19]
Amjadi et al. (2014)	Silver nanoparticle/MWCNT composites	2–14 (70%)	[25]
This work	Silver nanowire/polyethylene oxide	100 (50%)	-

## Data Availability

Data supporting this study are available within the article.
